# Considering the Performance Study of ZnO Nanofluid at Different Concentrations for the Full-Spectrum Utilization System

**DOI:** 10.1021/acsomega.4c10744

**Published:** 2025-04-16

**Authors:** Yangjie Zhuang, Yizhi Tian, Min Li

**Affiliations:** † College of Electrical Engineering, Xinjiang University, Urumqi 830017, P.R. China; ‡ Key Laboratory of Oasis Ecology of Education Ministry, College of Ecology and Environment, Xinjiang University, Urumqi 830017, P.R. China

## Abstract

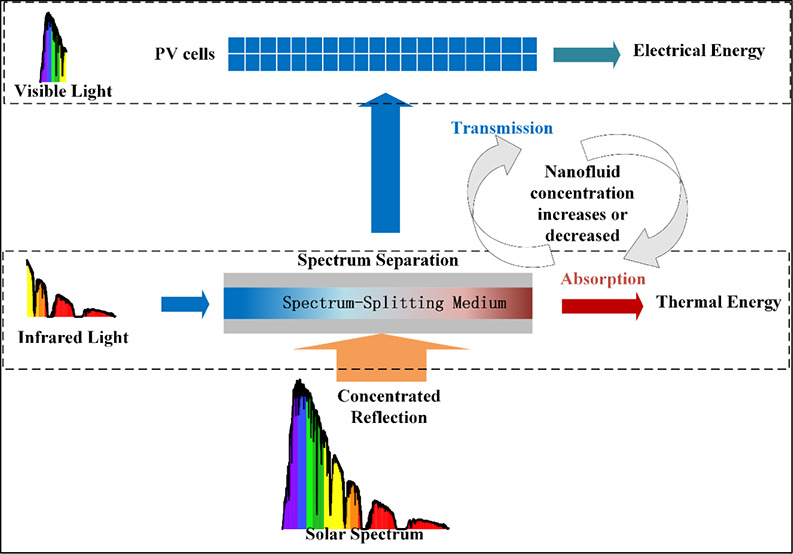

Single photovoltaic (PV) and photothermal (PT) technologies in solar energy applications are limited to the conversion of visible light and high-quality infrared spectra, respectively; this limitation results in relatively low energy utilization efficiency. In contrast, liquid spectrum-splitting technology enables the separation and conversion of various spectral bands, with the composition of the medium playing a pivotal role in the efficient utilization of the full spectrum. Compared to previous static spectral-splitting systems, this study introduces a dynamic nanofluid concentration control mechanism, which actively balances PV and PT contributions based on real-time solar conditions, achieving higher adaptability and efficiency. This study proposes a concentrated photovoltaic-thermal (CPVT) system based on the variable concentration of spectrum-splitting media, employing a concave-bottom, hollow pipeline structure. By introducing nanofluids with varying concentrations, we measured and analyzed spot uniformity and transmittance, comparing the system’s absorption properties for infrared light to its transmittance properties for visible light. A tunable model for photoelectric and photothermal–electric conversion was constructed, enabling the evaluation of differences in thermal and electrical performance. The results indicate that, within the designed spectrum-splitting pipeline, increases in nanofluid concentration correlate with improvements in both temperature and thermal efficiency. However, the photovoltaic efficiency decreased at higher concentrations. When the concentration reached approximately 280 ppm, the system achieved a peak comprehensive efficiency of 50.63%, demonstrating its superiority in adaptability and full-spectrum utilization compared to previous CPVT systems. As the concentration increased to 420 ppm, light transmittance nearly approached zero, resulting in a peak in thermal efficiency. This variability in concentration endows the system with a flexible capacity to modulate both thermal and electrical outputs, offering significant potential for further development.

## 1 Introduction

In the context of increasing energy demands, the gradual depletion of conventional energy sources, and ongoing environmental degradation, it is widely recognized that renewable energy will serve as a primary energy source in the future.^1^ Among these, the harnessing of solar power has attracted significant attention. Traditional photovoltaic-thermal (PVT) power generation systems exhibit low effectiveness in capturing both thermal and electrical energy. However, most existing spectrum-splitting systems mechanisms depend on static designs that cannot adjust to real-time solar irradiance or load requirements, restricting their efficiency and adaptability. The introduction of spectrum-splitting power generation systems mitigates the adverse effects of high temperatures on monocrystalline silicon cells, while also separating solar radiation to enhance the efficiency of entire spectrum energy utilization.^2^ The use of PVT technology for solar energy conversion has emerged as a viable solution. The performance of CPVT systems is directly affected by the geometric optical characteristics of the collector and the spectrum-splitting receiver.^3^ A compound parabolic concentrator (CPC) system introduced an interpolation method to optimize optical efficiency and the uniformity of concentrated irradiation, resulting in significant improvements in electrical and thermal performance. Parabolic solar collectors are widely used in commercial thermal power generation systems. A new hexagonal thermal receiver design surrounding the concentrator demonstrated, through experimental results, that optimizing the light path and spot uniformity of concentrated solar radiation can enhance solar cell performance.^4^ In contrast, this study incorporates dynamic nanofluid concentration control into a conventional parabolic trough concentrator system, addressing the limitations of static designs by enabling real-time adaptability to irradiance and load fluctuations. In these systems, nanofluids serve as conventional heat transfer media, capable of absorbing high-grade thermal infrared spectra while transmitting visible light that effectively interacts with photovoltaic cells. Therefore, the thermal properties of nanofluids are crucial to the system’s functionality, with factors such as temperature variations, nanoparticle concentration, and base fluid properties influencing performance.^5^ Nanofluid filters improve the performance of concentrated photovoltaic/thermal (CPVT) systems by possessing favorable thermodynamic properties and being adjustable through variations in nanoparticle content or size, which regulate the filtering rate and extinction coefficient. Reference^6^ introduced an HPV/D system based on nanofluid spectrum-splitting technology, demonstrating that increasing nanofluid concentration enhances freshwater yield and thermal efficiency. However, previous studies have primarily focused on fixed nanofluid concentrations, whereas this work introduces a dynamic control mechanism to optimize transmittance and absorption properties in response to changing conditions. At higher concentrations, photovoltaic efficiency decreases, while the thermal-to-electrical ratio increases. This control enables adaptation to seasonal and climatic variations across regions, allowing rapid responses to the diverse energy demands of different users.^7^ Therefore, fluctuations in nanofluid concentration lead to variations in PVT system efficiencies. Furthermore, the flow characteristics of the conduit impose substantial constraints on the heat transfer properties of nanofluids, necessitating careful consideration of the integrated performance metrics of microchannels with different shapes.^8^ The remaining visible light, after passing through the nanofluid conduit, reaches the photovoltaic cell surface, with a substantial portion of solar energy converted into waste heat. As spectrum-splitting technology advances, efficient solar cells that are not affected by temperature increases can help mitigate this issue. Compared to other cells, gallium arsenide concentrator cells exhibit minimal temperature-induced effects on open-circuit voltage, with a reduction rate of 5.50 mV/°C.^9^ Additionally, by developing efficient thermal management technologies, reducing heat loss, and recovering residual heat, this issue can be further alleviated.^10^

Building upon previous research, this study designs the geometric structure of the device receiver under the conditions of a traditional parabolic trough concentrator, aiming to enhance the uniformity of the light spot on the concentrator cell. By integrating dynamic nanofluid concentration adjustment, the system achieves superior adaptability compared to static spectrum-splitting designs, allowing for optimized PV and PT outputs across varying irradiance and load scenarios. Through fluid heat recovery, the study aims to control the transmittance of visible light spectra, thereby modulating both photoelectric and thermal efficiency, and explores a novel approach for the efficient full-spectrum utilization of solar energy. Table 1 compares the proposed dynamic nanofluid concentration system with previous spectrum-splitting technologies, highlighting its advantages in adaptability, energy utilization efficiency, and system stability.

**Table 1:** Comparison between the Proposed Dynamic Nanofluid Concentration System and Previous Spectrum-Splitting Technologies

Feature	Previous Systems	Proposed System
Nanofluid concentration control	Fixed or absent; limited adaptability to real-time changes.	Dynamic adjustment based on solar irradiance and load demand, enhancing flexibility and efficiency.
Energy utilization efficiency	Lower due to static designs and energy mismatches.	Integrated with dynamic concentration control for optimized full-spectrum energy utilization.
System stability	Output fluctuations due to irradiance changes.	Higher efficiency achieved by real-time optimization of PV and thermal outputs.
Adaptability to conditions	Limited to specific climates or static load patterns.	Broad adaptability across varying climates and load demands.

## 2 System Principle and Structural Design

### 2.1 Design Principle of the Concentrated Spectrum-Splitting System

**Figure 1:**
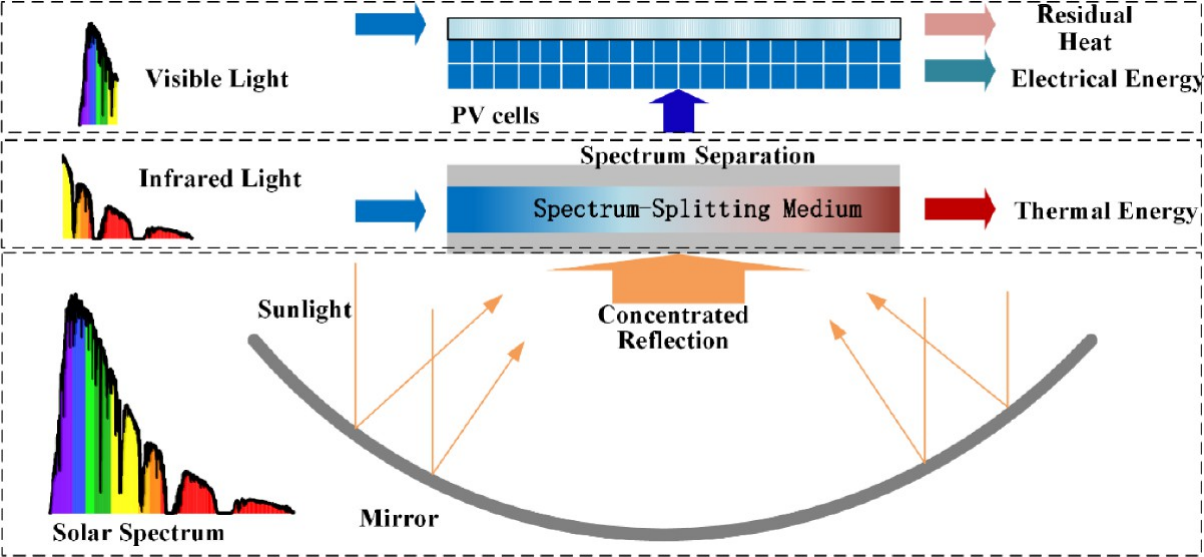
Mechanism of Spectral Energy Utilization in the PCS System.

The mechanism of spectral energy utilization^11^ in the parabolic trough-based Concentrated Spectrum-Splitting System (PCS) is illustrated in Figure 1. The principle of liquid spectrum-splitting media for absorbing infrared spectra has been extensively applied in various concentrator configurations. The PCS system first focuses high-energy-density solar radiation using a reflector. The sunlight then traverses the spectrum-splitting pipeline, whose geometric design ensures optimal spot uniformity on the photovoltaic (PV) surface, thereby increasing power generation efficiency. The pipeline is also filled with a spectrum-splitting medium of specific concentration, allowing for minimal light refraction but varying absorption characteristics based on the medium’s concentration, with the absorbed infrared spectra converted into internal energy. Finally, the remaining visible light reaches the photovoltaic cell surface for photoelectric conversion. The cooling conduit behind the photovoltaic cell is filled with coolant, which absorbs the residual heat from the cell. By adjusting the concentration of the spectrum-splitting medium, the system can regulate both light absorption within the pipeline and light transmittance. As a result, the amount of light reaching the PV cell surface varies, allowing for flexible and effective control of power output.

**Figure 2:**
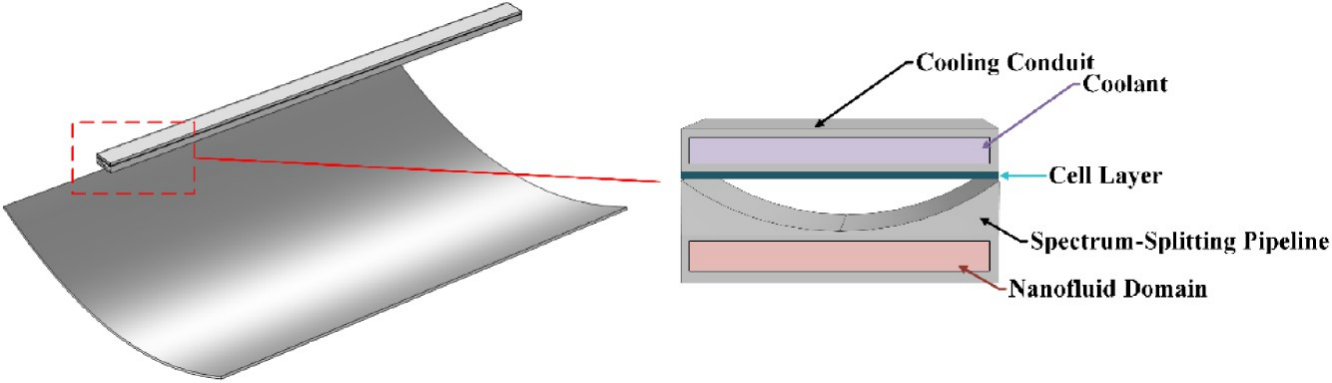
Structure of the Concentrated Spectrum-Splitting Solar Energy Utilization System.

The concentrated spectrum-splitting system comprises a parabolic trough collector mirror, spectrum-splitting pipeline, spectrum-splitting medium, concentrated photovoltaic cell, cooling conduit, and cooling water, as shown in.^1^ In Figure 2, the concentrator mirror is placed at the bottom layer, with the spectrum-splitting pipeline positioned above it. The pipeline is a hollow structure with a flat base and concave upper surface, filled with nanofluid. The liquid spectrum-splitting medium absorbs solar radiation, increasing its temperature, which facilitates thermal energy storage following heat exchange. This thermal energy can also be converted into electrical energy by a power generation system. The top layer comprises the concentrated photovoltaic cell, which absorbs transmitted visible light and generates electrical energy. The system is designed based on the optical energy variation characteristics of the spectrum-splitting medium concentration.

### 2.2 Model Design

#### 2.2.1 Concentration Process

The model employs an Al_2_O_3_ mirror, offering advantages such as high reflectivity, corrosion resistance, high-temperature durability, and excellent optical transparency. The parameters are specified in Table 2.

**Table 2:** Basic Properties of the Concentrator Mirror and Pipeline

Properties	Parameters	Values	Units
Mirror absorption coefficient	∼	0.10	1
Mirror surface roughness	σ_1_	1.75	mrad
Maximum disk Angle of the sun	δ_s_	32′	∼
The focal length of the mirror	*f*	0.40	m
Receiver width	*D*	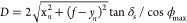	m
Maximum concentration ratio	CR	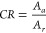	∼
Light projection area	*A*_a_	0.58	m^2^
Receiving area	*A*_r_	0.03	m^2^
Glass transmittance	∼	0.98	1
Nanofluid thickness	*L*_m_	10	mm

The spectrum-splitting pipeline has a hollow structure with a flat bottom and concave cylindrical upper surface. The incident light side is flat, while the opposite side is curved. Considering the effect of the pipeline’s cross-sectional shape on light refraction, the concentrated light is refracted through the concave cavity to form relatively collimated parallel rays. Supporting Information S1 gives the specific shape of the cross-section.

The nanofluid pipeline utilizes an all-glass vacuum tube, with the outer vacuum layer modeling omitted. Quartz glass is employed as the material, exhibiting strong absorption characteristics for short-wavelength light, particularly around 300 nm, and demonstrating excellent transparency across the entire visible spectrum.

To validate the accuracy of the ray-tracing model, a 3D model of the mirror and spectrum-splitting pipeline was constructed using the parameters described above in the optical software TracePro. The light source intensity was set to 1000 W/m^2^, and 20,000 rays were emitted. Since variations in the concentration of the spectrum-splitting medium primarily influence light absorption, this model does not consider its refractive effect on the light path. Supporting Information S2 is given in the TracePro software based on the light aggregation and the initial simulation diagram of the model, and the subsequent simulation of light tracing will be based on this model.

Spot uniformity is employed to quantify the uniformity of solar radiation energy received on the photovoltaic cell surface, representing the ratio of the maximum irradiance intensity to the average irradiance intensity on the receiving surface:^12^
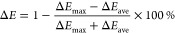
1

The study investigates the effect of the curvature of the concave side of the spectrum-splitting pipeline on the spot distribution received by the cell surface.

**Figure 3:**
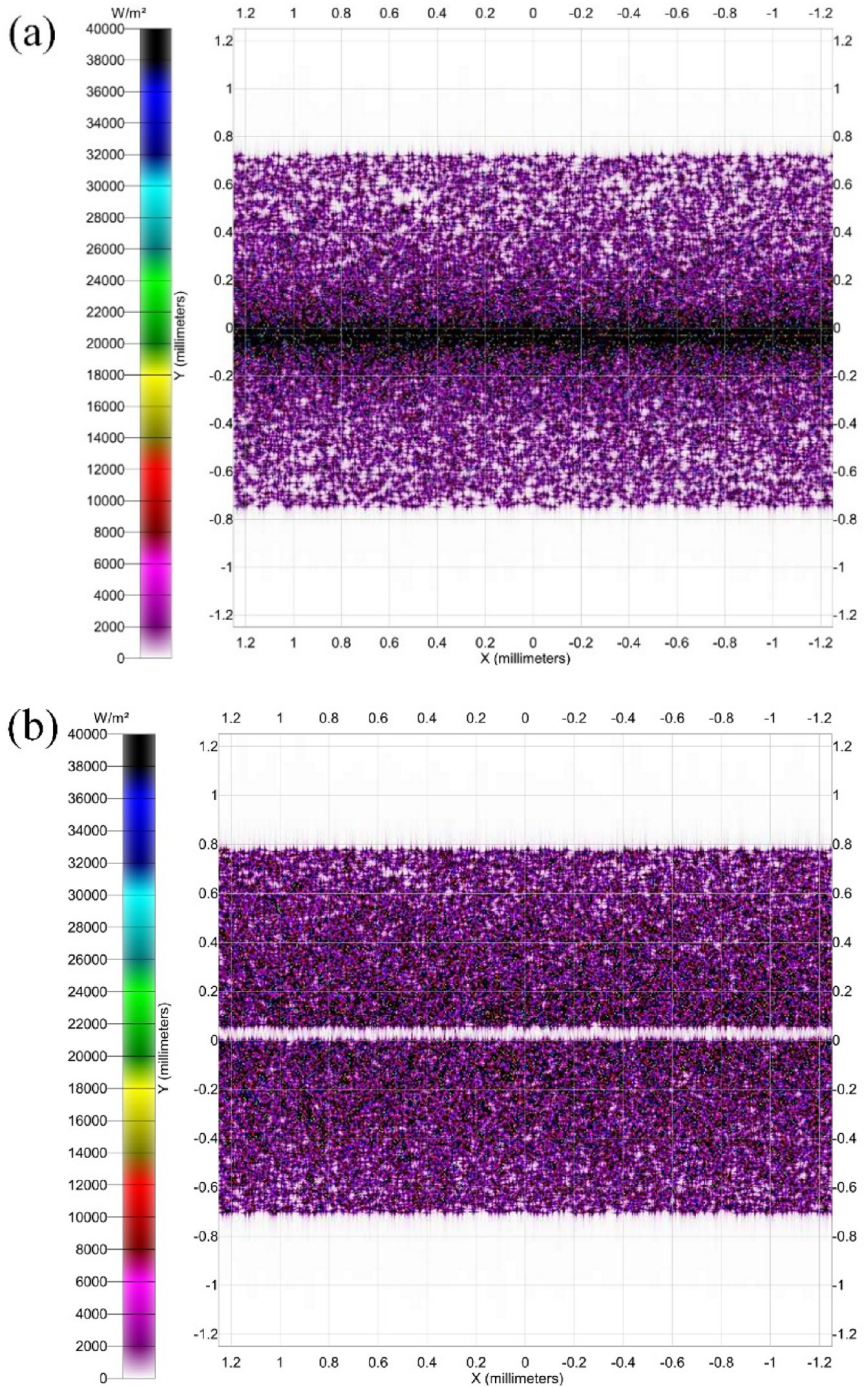
(a) Spot on the cell surface with direct concentration. (b) After light uniformity adjustment through the pipeline.

The integrated optical homogenizers used in the literature^13^ to improve the solar flux distribution of CPV cells, while more homogeneous in the middle, produce severely inhomogeneous flux distributions near the corners and edges of the cells and are more complicated to equip, compared to direct concentrators. As shown in^14^Figure 3 and Table 3, the extreme inhomogeneity can be reduced by using a beam-splitting duct, and considering the effect of curvature on the optical performance of the concave surface, the homogeneity reaches a maximum value of 28.41% when the curvature is reduced to 1.19, which is a 14% improvement in homogeneity. However, further reduction of the curvature leads to a decrease in the uniformity. This suggests that the new beam-splitting pipe greatly improves the spot uniformity on the surface of the concentrator cell, which is simple in design and serves as both a homogenizer and a container.

**Table 3:** Optical Performance of the Spectrum-Splitting Pipeline with Different Curvatures on the Concave Side

Curvature on the Concave Side	Maximum Irradiance Intensity (W/m^2^)	Average Irradiance Intensity (W/m^2^)	Spot Uniformity Δ*E*
1.28	297,708	48,075	27.81%
1.25	298,948	48,899	28.12%
1.22	298,763	49,218	28.29%
1.19	297,177	49,209	28.41%
1.16	297,600	49,205	28.38%
1.13	306,819	49,179	27.63%
1.11	306,697	49,174	27.63%

#### 2.2.2 Spectrum-Splitting Medium

Nanofluids, as spectrum-splitting media, exhibit distinct optical properties. The application of nanofluids in PVT systems not only facilitates the separation of PV and PT units but also improves the overall performance and reliability of the system, thus advancing the development and application of solar energy technologies. Glycerol-based ZnO nanofluids demonstrate excellent physical stability and cost-effectiveness, with ZnO nanoparticles showing superior dispersibility across various concentrations, a high specific heat capacity, and favorable thermal conductivity. Based on the analysis of the physical properties of various spectrum-splitting media in the literature,^14^ this study selects ZnO nanofluid as the spectrum-splitting medium.

**Figure 4:**
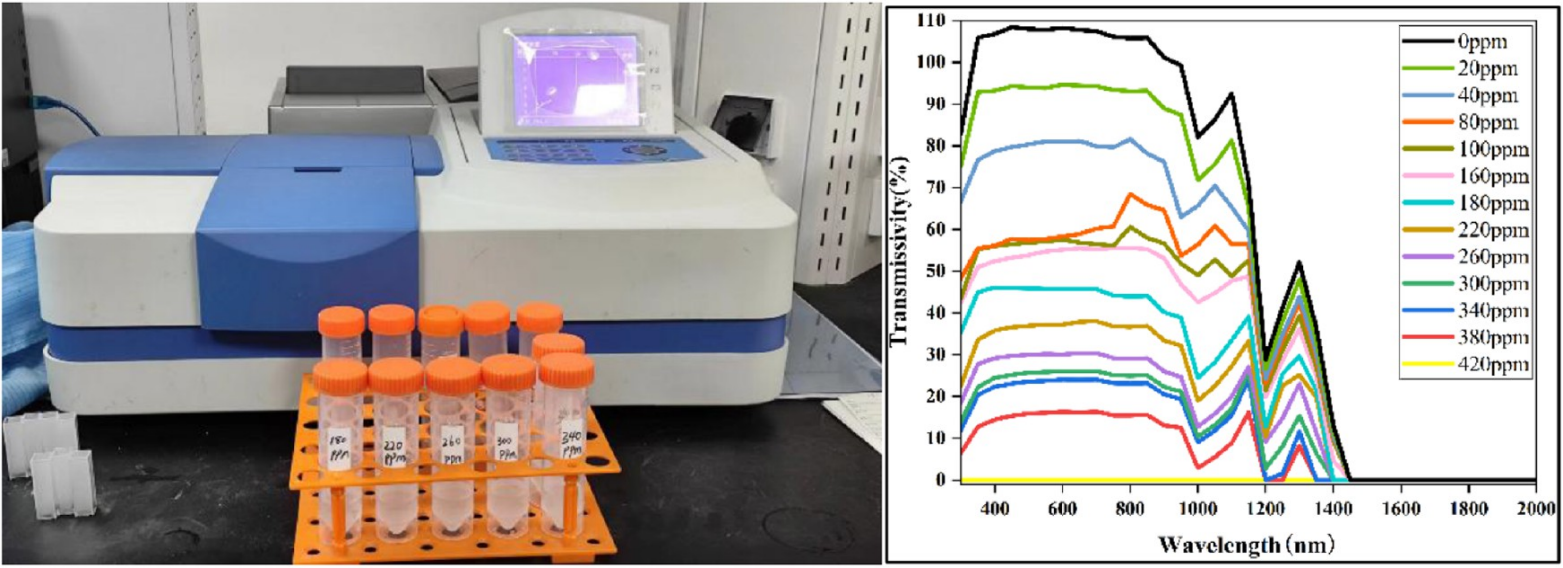
Transmittance of nanofluids at different concentrations measured by the spectrophotometer.

The solar spectrum range examined in this study extends from 300 to 2500 nm. Nanoparticles with varying mass concentrations were prepared, and the transmittance and absorbance (abs) of their solutions were measured using a spectrophotometer. Specific preparation procedures with data plots of absorbance are given in the Supporting Information S3,S4. The transmittance of light at different wavelengths and concentrations was subsequently plotted, as illustrated in Figure 4. ZnO nanofluids exhibit high spectral transmittance in the 400–1100 nm range and high spectral absorbance in the 1500–2500 nm range. As concentration increases, the spectral transmittance of the nanofluid gradually decreases in the 400–900 nm range, whereas in the 1400–2500 nm range, the transmittance of ZnO nanofluids at various concentrations approaches zero. The trend in transmittance variation corresponds with the system’s operational requirements for controlling the concentration of the spectrum-splitting medium.

The model utilizes the Rayleigh scattering model^15^ to evaluate the optical properties of the nanofluid. In this experiment, the average particle size of the nanoparticles is relatively small, well below the wavelength of the incident radiation, with pure liquid serving as the base fluid and a nanofluid volume fraction of less than 0.6%. Therefore, scattering effects can be neglected, and only the absorption effect is considered. The ideal optical properties for spectrum splitting in this study are as follows.

The calculation formula for the extinction coefficient as a function of volume fraction is given as follows:
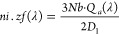
2

This formula applies to dilute dispersed systems, assuming negligible interactions between particles. The extinction coefficient *ni*_total_(λ) indicates how the absorption of incident light varies with the wavelength λ of the light:
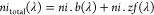
3

The extinction coefficient *ni*_total_(λ) consists of the extinction coefficient of the base fluid *nib* (λ) and the extinction coefficient of the nanoparticles *ni.zf* (λ).

Since the extinction coefficients for both the spectrum-splitting pipeline and the nanofluid are high for both short and long wavelengths, it is assumed that this portion of the spectrum is fully absorbed. Based on the Beer–Lambert law and the absorption coefficient, the light absorption rate by the nanofluid with thickness *L*_m_ in the direction of incident light, as well as the absorption rate and transmittance of the spectrum-splitting pipeline, can be calculated as follows:
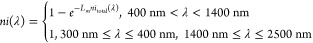
4
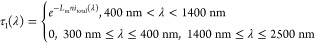
5

#### 2.2.3 Concentrated Photovoltaic Cell Section

**Figure 5:**
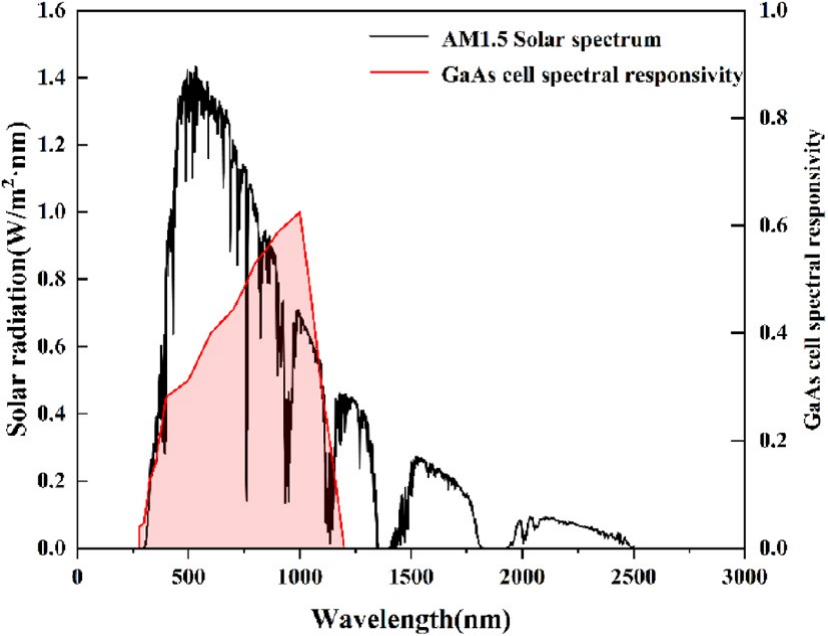
AM1.5 solar spectrum and spectral response curve of the gallium arsenide (GaAs) cell.

Gallium arsenide (GaAs) cells offer distinct advantages in CPV systems. First, these cells are designed to function efficiently under concentrated light conditions. Second, their electrical performance parameters exhibit reduced sensitivity to temperature fluctuations when subjected to such conditions. Within a certain range of light intensity, as the irradiance on the photovoltaic cell increases, the cell temperature rises, thereby reducing efficiency. However, compared to the magnitude of the temperature increase, the efficiency drop in GaAs photovoltaic cells is relatively small, underscoring their advantage over conventional photovoltaic cells in terms of resilience to temperature fluctuations. Figure 5 illustrates the AM1.5 solar spectrum and the spectral response curve of the GaAs cell, demonstrating that GaAs cells effectively perform photoelectric conversion within the 400–1000 nm wavelength range of sunlight.

The water-filled flow channel, acting as the coolant, is in direct contact with the thermally conductive adhesive applied to the back of the photovoltaic cell. The photovoltaic cell converts a portion of the received irradiation into electrical energy, with a smaller fraction being converted into thermal energy. The generated heat is absorbed by the cooling fluid, thereby reducing the temperature rise of the photovoltaic cell.^17^

### 2.3 Numerical Model

A 3D physical model of the PCS was constructed using COMSOL simulation software to validate the simulation results for photoelectric and photothermal-electric coupling. To simplify the numerical model of the proposed hybrid system, the following assumptions were made in this study:

1. Simulation assumptions

Steady-state operation: We assumed the system operates under steady-state conditions, which is a common assumption in thermodynamic and photovoltaic system simulations. This simplifies the modeling of the system’s energy conversion processes, by neglecting transient thermal effects. Setting and measurement of the relevant thermal properties of nanofluids are detailed in the Supporting Information.

Ambient temperature: The ambient temperature was set to 293.15 K (20 °C), which is a typical average temperature used in solar energy studies to simulate realistic operating conditions. This value was chosen to approximate standard operating conditions in solar energy systems, where extreme temperature variations are less likely.

Ideal nNanoparticles: Zinc oxide (ZnO) nanoparticles were assumed to be spherical with a uniform diameter of 20 nm. This assumption is based on the simplicity of the model and the available literature,^23^ which often assumes idealized properties to isolate the main factors influencing performance.

2. Environmental conditions

Solar irradiance: The light emission irradiance was set to 1000 W/m^2^, which corresponds to the standard solar irradiance at the Earth’s surface under clear sky conditions (AM1.5 spectrum). This value is consistent with typical solar energy applications and was used to model the system’s maximum potential performance.

Solar spectrum: The simulation used a wavelength range from 300 to 2500 nm to cover the complete solar spectrum, as this range is relevant for both photovoltaic and photothermal conversion. The simulation parameters ensure that the system optimizes the absorption of both visible and infrared light, enabling full-spectrum utilization.

3. Properties of materials

Nanofluid properties: The nanofluid, specifically glycerol-based ZnO nanofluid, was chosen based on its favorable thermal stability, high dispersibility, and suitable absorption characteristics in the infrared range. Physical properties such as thermal conductivity and specific heat capacity were approximated by fitting published data and measurements of ZnO nanofluids at specific concentrations.

Material selection: The choice of materials (e.g., gallium arsenide photovoltaic cells, aluminum oxide mirrors, and zinc oxide nanoparticles) was made to optimize both electrical and thermal efficiencies. These materials are known for their high efficiency in concentrated photovoltaic/thermal systems, and their properties are well-established in the literature.^24,25^

4. Simulation model and software:

COMSOL simulation: The COMSOL Multiphysics software was used to simulate the coupled heat transfer and optical processes. The software’s geometric optics and solid–fluid heat transfer modules enabled us to model the interactions between light, nanofluid, and the photovoltaic cells. The model was designed to ensure an accurate representation of the system’s energy conversion and heat management under varying concentrations of nanofluid and irradiance levels.

5. Concentration of nanofluid

The concentration of the nanofluid (Nb) was varied within a realistic range (0 ppm to 420 ppm) based on literature values and experimental results from similar studies. This range allows the model to evaluate the system’s performance across typical operating conditions and to explore the effects of nanofluid concentration on both electrical and thermal efficiencies.

**Figure 6:**
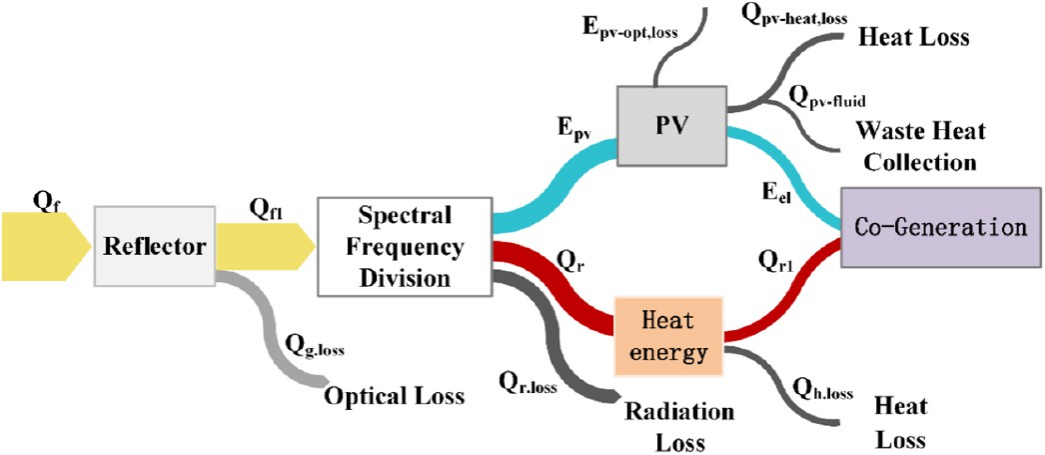
System energy flow model.

Based on the mathematical models for each subsystem design and considering the characteristics of energy conversion, storage, and transmission within the system, an analysis of the energy transfer process was performed. Models were developed to analyze photoelectric conversion efficiency, photothermal conversion efficiency, and nanofluid temperature in the concentrated spectrum-splitting solar energy utilization system. The energy transfer model of the system is illustrated in Figure 6.

#### 2.3.1 Process of Concentrated Reflection

Solar radiation simulation is typically based on the fundamental principles of blackbody radiation and solar characteristics. This involves simulating the sun’s temperature, spectral distribution, and radiation intensity. The energy spectral density of blackbody radiation is given by
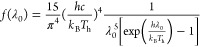
6

Here, λ_0_ is the wavelength within the designated system bandwidth of 300 to 2500 nm, and *T*_h_ represents the blackbody temperature.

In the concentration process, the full solar spectrum falls onto the reflector and is subsequently directed to the frequency-splitting conduit. The solar energy received in the concentrated area, and the optical losses of the reflector can then be calculated as follows:
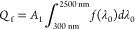
7
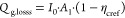
8

Here, *I*_0_ denotes the direct normal irradiance, and *η*_cref_ is taken as 0.98.

#### 2.3.2 Process of Photovoltaic Conversion

Energy reaching the surface of the photovoltaic cell:
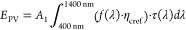
9

The optical losses generated during the energy conversion process of the photovoltaic cell can be expressed as
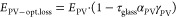
10

Here, γ_PV_ is related to the intrinsic properties of the photovoltaic module. This study employs a single-junction thin-film GaAs photovoltaic cell to convert solar energy into electrical energy. The power balance of the photovoltaic cell is expressed as^18^
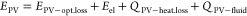
11
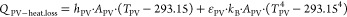
12
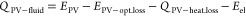
13

Energy converted into electrical power:
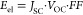
14

The spectrum transmitted through the nanofluid is transformed into electrical energy by the photovoltaic cell. The electric efficiency is computed using the formula:
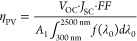
15

#### 2.3.3 Thermal Model of the Frequency-Splitting Subsystem

A steady-state model is used for the analysis of the system. The optical properties of the optical fluid, cooling water, cover glass, aluminum plate, and air gap are assumed to be temperature-independent. The temperature differential between the optical fluid and cooling water along the solar radiation path is neglected. In this study, an energy balance equation is employed for the system. The energy balance equations for the optical fluid, PV cell, and coolant are considered in the calculations, enabling the determination of the system’s thermal efficiency. The energy conservation equation governing the spectrum-splitting process is as follows:
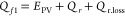
16
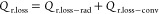
17

The radiative and convective losses of the nanofluid are expressed as
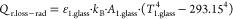
18
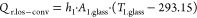
19

Here, *Q*_r.loss–rad_ and *Q*_r.loss–conv_ represent the radiative losses to the surroundings. Thus, the thermal energy acquired by the frequency-splitting duct is
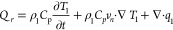
20
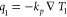
21

The physical properties of the mixed liquid are derived from the uniform distribution of nanoparticles and glycerol. The assumed fraction for converting thermal energy into electrical energy can be obtained according to^20^
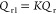
22

In the formula, *K* represents the conversion fraction, with the assumption that thermal energy is transformed into electrical energy through a Carnot cycle, which yields the maximum power derivable from thermal energy. The system’s thermal efficiency can be determined from the solutions of these equations as follows:
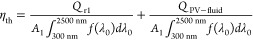
23

#### 2.3.4 System Efficiency Calculation

Thermal output and electrical output are distinct in value. Therefore, the total energy efficiency of the system is calculated as follows:^21^
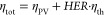
24

Here, *HER* is a weighting factor that reflects the value of thermal energy relative to electrical energy,^22^ known as the heat-to-electricity ratio.

## 3 Results and Discussion

### 3.1 Analysis of Optical Results

To validate the system’s utilization of the full spectrum, this study analyzes variations in light and thermal characteristics across different materials and investigates the operating mechanisms of photoelectric and photothermal-electric coupling under varying nanofluid concentrations. The system is simulated using coupled ray tracing in COMSOL software. The model incorporates modules for geometric optics, solid and fluid heat transfer, and peristaltic flow, coupled to generate a ray-based heat source and nonisothermal flow in a Multiphysics field, thereby facilitating model resolution.

**Figure 7:**
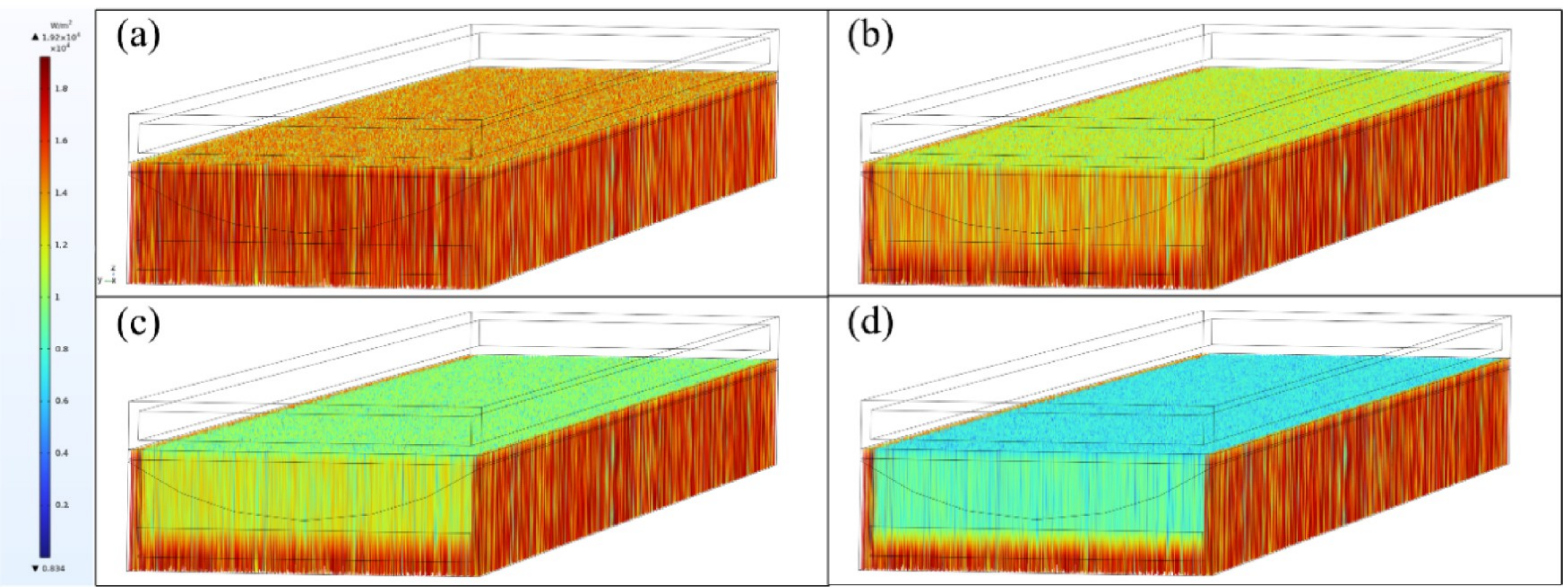
Variation in light intensity attenuation: (a) 0 ppm; (b) 40 ppm; (c) 100 ppm; and (d) 220 ppm.

**Figure 8:**
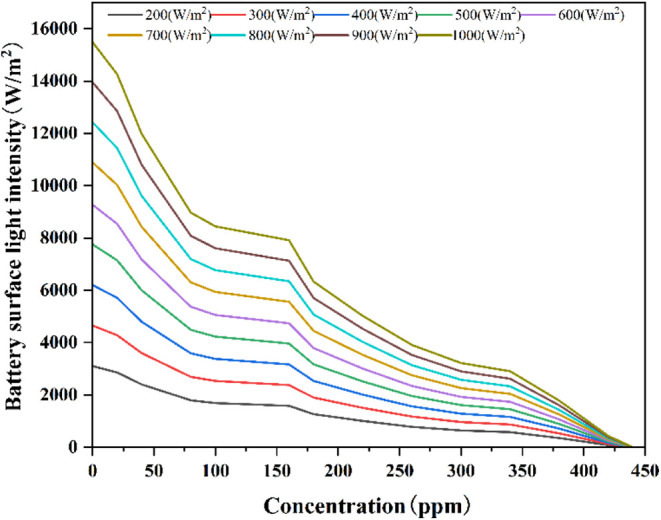
Effect of concentration variation on cell surface light intensity under different irradiance levels.

The light emission irradiance is set at 1000 W/m^2^, with wavelengths ranging from 300 to 2500 nm and a geometric concentration ratio of 20. The light propagates in the specified direction, reaching an intensity of 1.92 × 10_4_ W/m^2^ before entering the frequency-splitting duct. Details of light passing through the frequency-splitting duct and reaching the concentrator cell are presented in Figure 7a–d, illustrating light attenuation at nanofluid concentrations of 0, 40, 100, and 220 ppm, respectively. Higher concentrations result in significant light attenuation as light passes through the frequency-splitting nanofluid region; some rays at the edges pass through the lens with less attenuation, and a small portion of light escapes. Ultimately, the main beam reaches the cell surface, with the cell’s rear cooling conduit located above it. As the light is confined to the cell layer, the cooling conduit remains unaffected by light. Figure 8 illustrates the effect of concentration variation on cell surface light intensity under different irradiance levels. As shown, when concentration increases from 0 to 80 ppm, the light intensity at the cell surface exhibits a rapid attenuation under varying irradiance levels, with more pronounced effects at higher irradiance. Subsequently, light intensity decreases linearly with concentration variation. This indicates that as the concentration increases, the extinction coefficient of the nanofluid increases, improving its ability to absorb light. When the concentration reaches 420 ppm, the light intensity is almost reduced to zero. The effect of nanofluid concentration on light transmittance has been well-documented in similar studies. Yasmin et al.^23^ observed similar reductions in light transmittance with increasing nanoparticle concentration in nanofluid-based systems, particularly when the concentration surpassed certain thresholds. Thus, this model allows for the analysis of the impact of variations in solar irradiance and nanofluid concentration on light intensity, providing a basis for further investigation into the photoelectric conversion characteristics of the concentrated frequency-splitting solar system.

### 3.2 Thermodynamic Analysis Results

**Figure 9:**
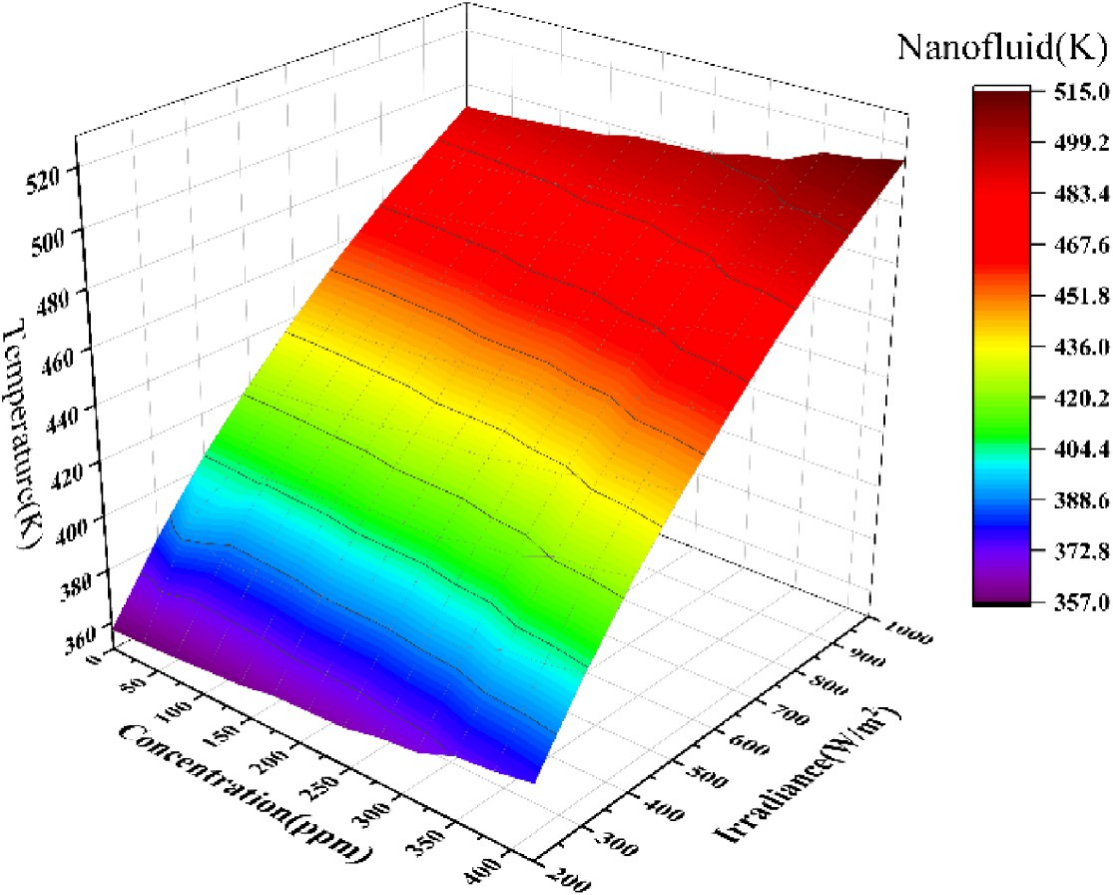
Nanofluid temperature variation.

**Figure 10:**
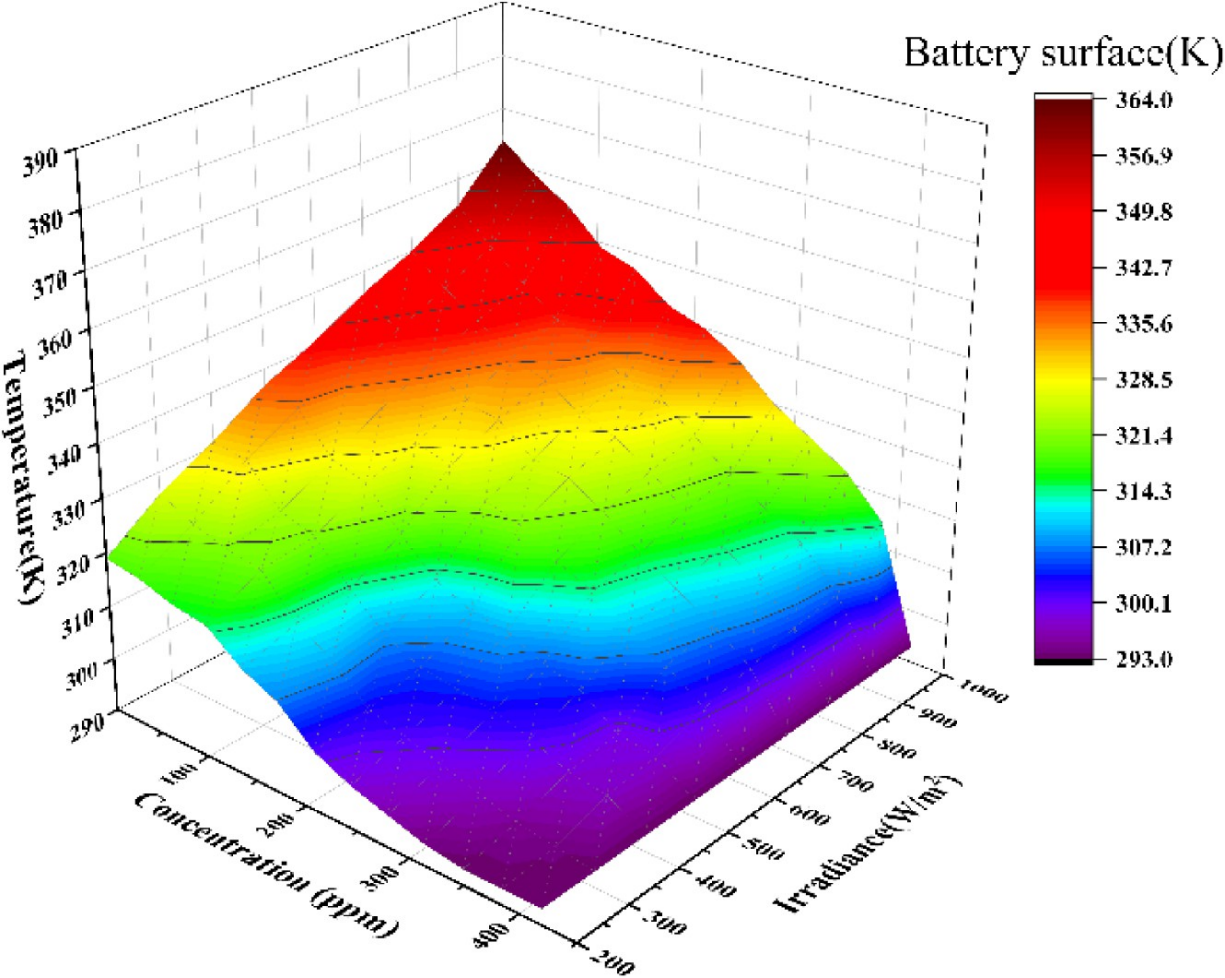
Cell temperature variation.

Based on the optimal nanofluid flow rate determined from the aforementioned three-dimensional model, an analysis of various parameters was conducted. Figure 4 illustrates the changes in spectral transmittance at an optical thickness of 10 mm. Under a 20-fold concentration ratio, the system’s output temperature was analyzed in relation to variations in solar irradiance and nanofluid concentration, with results presented in Figure 9. As the light concentration increases, the nanofluid temperature rises due to enhanced absorption by the internal particles. The increase in nanofluid temperature with concentration and its effect on cell temperature aligns with findings in the study by Abdelrazik et al.,^26,27^ who observed a similar trend in their investigation of nanofluid-based cooling systems for solar applications. Under the conditioning of the Supporting Information S5 about the optimal flow rate, the evaluation of the temperature of the nanofluid is carried out. At an irradiance of 200 W/m^2^, the temperature increases by 20 K as the nanofluid concentration varies from 0 to 420 ppm. At an irradiance of 1000 W/m^2^, the temperature increases by nearly 25 K. It is evident that as concentration increases, more heat in the 400–1400 nm wavelength band is absorbed. Meanwhile, as shown in Figure 10, the management of the mass fraction of zinc oxide nanofluid has a minor effect on the cell temperature. As the mass fraction increases, the temperature of the photovoltaic cell decreases significantly under varying irradiance levels, as most of the solar radiation is absorbed by the optical zinc oxide nanofluid, generating heat. At an irradiance of 1000 W/m^2^, the maximum cell temperature reaches 364 K at a concentration of 0 ppm.

At 0 ppm, the absorption of both shortwave and longwave components by the glycerol-based fluid and duct significantly reduces the low-grade thermal energy impact on the concentrator cell’s temperature coefficient, lowering the cell surface temperature by 372.61% compared to prefrequency-splitting conditions and mitigating the risk of thermal damage. The residual visible light and a small fraction of infrared light are absorbed during the frequency-splitting process as concentration changes, leading to a rapid decrease in cell surface temperature, especially at high irradiance levels.

### 3.3 Comprehensive System Performance Evaluation

**Figure 11:**
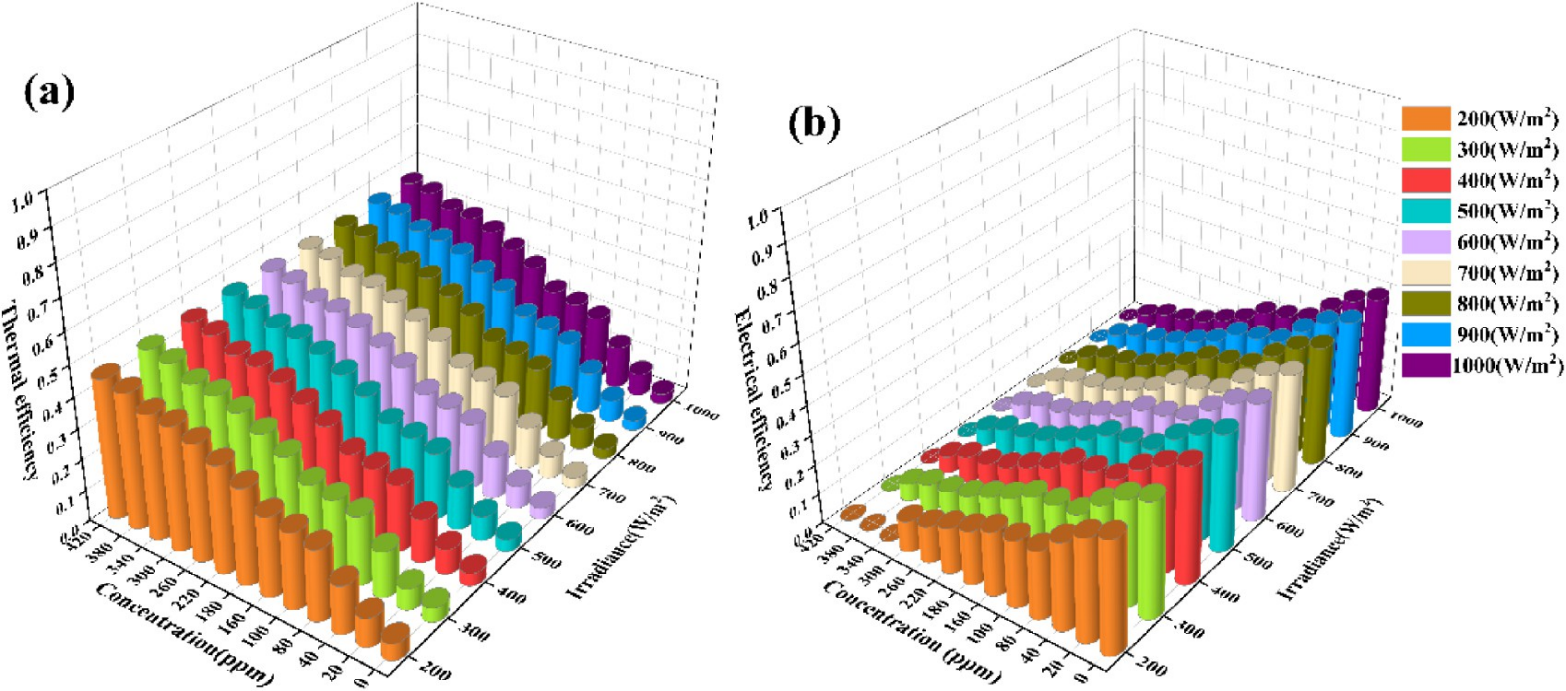
(a) System thermal efficiency. (b) System electrical efficiency.

Previous findings indicate that increasing the mass fraction of zinc oxide nanofluid raises the nanofluid temperature, reduces the amount of light incident on the cell surface, and lowers the surface temperature. Consequently, Figure 11 demonstrates the variations in thermal and electrical efficiency of the system under different irradiance conditions. At the same irradiance, as the mass fraction of nanofluid increases, each 40 ppm increase in concentration results in a 3% to 4% rise in thermal efficiency. However, after reaching 300 ppm, the rate of increase in thermal efficiency diminishes, with efficiency increasing by only 5% as the concentration rises from 300 to 420 ppm. Furthermore, at the same concentration, as irradiance increases from 200 W/m^2^ to 1000 W/m^2^, thermal losses increase, resulting in an approximate 2% reduction in overall thermal efficiency. The cooling conduit installed at the back of the photovoltaic cell to absorb excess heat results in an average increase in the system’s thermal efficiency by 1.05% compared to a system without heat absorption.

For photovoltaic cells, an increase in mass fraction from 0 to 100 ppm results in a decrease in cell efficiency by 11%. At this concentration, the transmission window of the zinc oxide nanofluid remains within the ideal energy window for the gallium arsenide cell, allowing solar radiation in the visible light range to continue contributing to power generation. As a result, as the mass fraction increases, the power output of the concentrator cell decreases, ultimately reaching zero at a high concentration of 420 ppm. Photovoltaic power generation efficiency varies with different irradiance levels, primarily due to variations in light intensity. At the same concentration, each 100 W/m^2^ increase in irradiance leads to a 0.12% to 0.40% improvement in generation efficiency. However, at concentrations below 40 ppm, the cell efficiency under higher irradiance of 1000 W/m^2^ is influenced by temperature, resulting in a 2% decrease compared to 900 W/m^2^.

**Figure 12:**
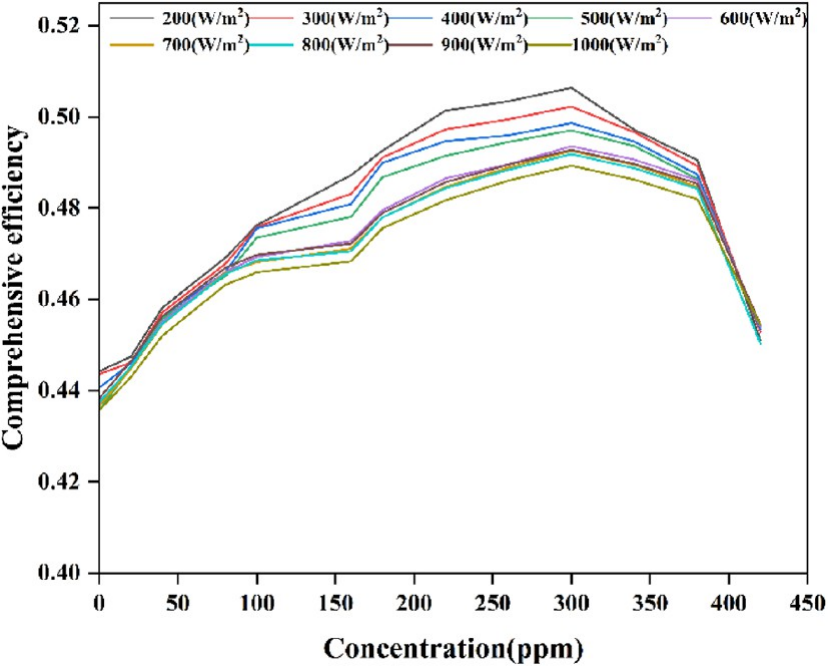
Comprehensive system efficiency.

The overall efficiency of the system in absorbing the solar spectrum is presented in Figure 12. Under varying irradiance levels, thermal efficiency increases significantly with rising concentration. The temperature effect on the photovoltaic cell diminishes; however, the intensity of the incident spectral light decreases, leading to a slight decline in power generation efficiency, while overall efficiency increases significantly. At approximately 280 ppm concentration, the overall efficiency reaches its peak. These findings are consistent with those of Rashid et al.,^28^ who studied the effects of nanofluid concentration on CPVT systems and observed a similar trade-off between thermal and electrical efficiency, with peak efficiency occurring at a specific concentration and decreasing as the concentration increased further. Though at the expense of some electrical efficiency, the frequency-splitting medium gradually attains higher temperatures with further increases in concentration. Once the thermal efficiency of the frequency-splitting medium reaches its maximum, it stabilizes, while the photovoltaic cell’s power generation efficiency significantly declines, resulting in a rapid decrease in overall efficiency. The Supporting Information S6 also provides the integrated electrical efficiency at different concentration ratios, showing that further increases in the concentration ratio lead to a gradual decrease in efficiency.

### 3.4 Experimental Verification

**Figure 13:**
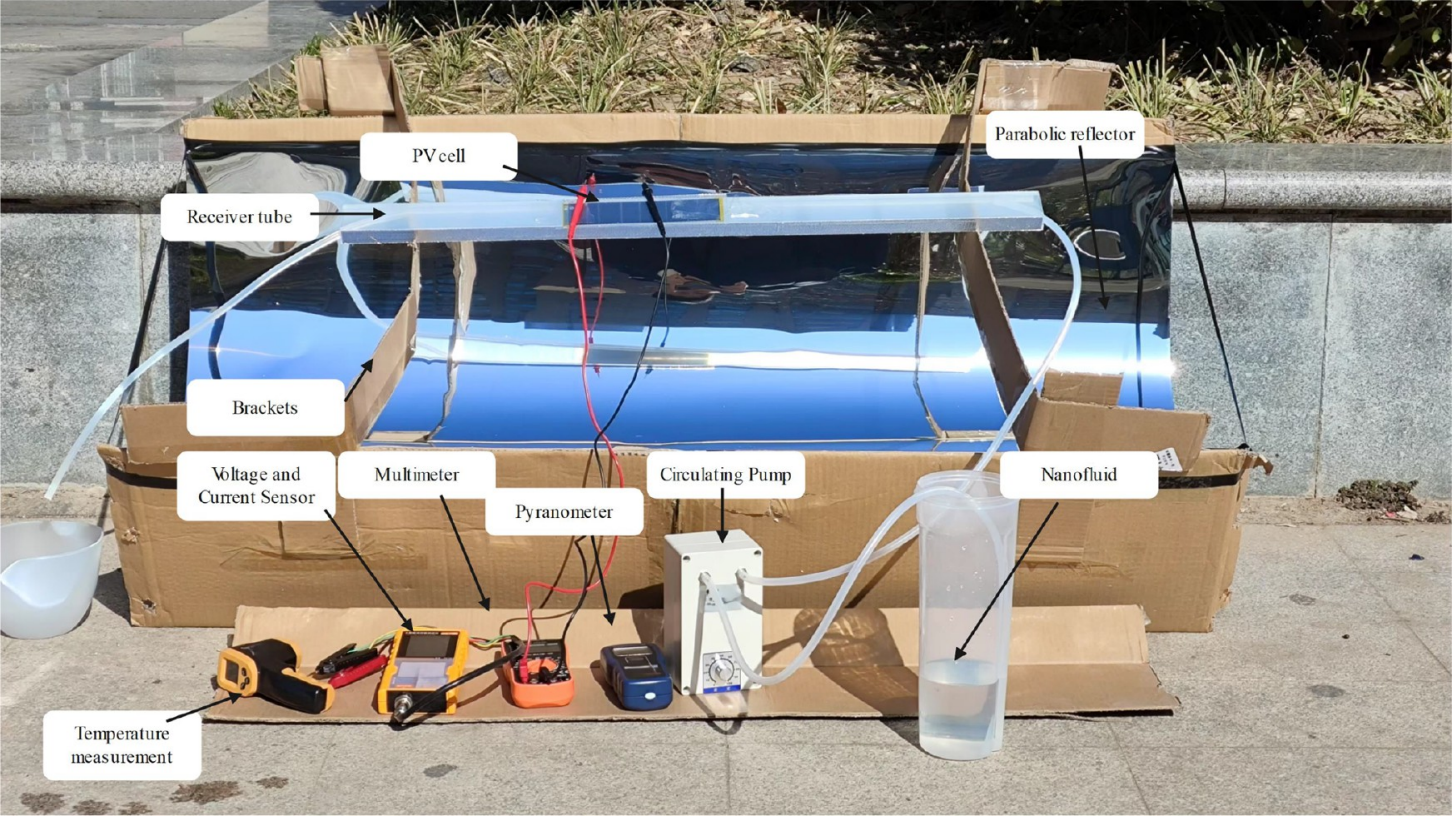
Experimental setup for the concentrating frequency division system.

The experimental setup is shown in Figure 13. The experiment was conducted over 4 days from January 22 to January 25 under sunny and cloudy skies. The mass flow rate for the experiment was set to the optimal flow rate for the simulation. The main objectives of the experiment were to measure the temperature of the nanofluid and the power output of the photovoltaic (PV) system once the nanofluid reached a steady state. Prior to this, the thermal conductivity and heat capacity of the nanofluid were also measured, provided by the Supporting Information S7, S8, which was used to support the smooth running of the experiment. SM206-SOLAR, RC9550 m was used to measure the solar radiation level and temperature variations, and PVT801 was used to capture the electrical properties of the PV panels. In this experiment, these values were recorded manually. These values will be recorded when the weather clears and the temperature increases. Refer to Supporting Information S9 for the specific device model used in the experiment.

**Figure 14:**
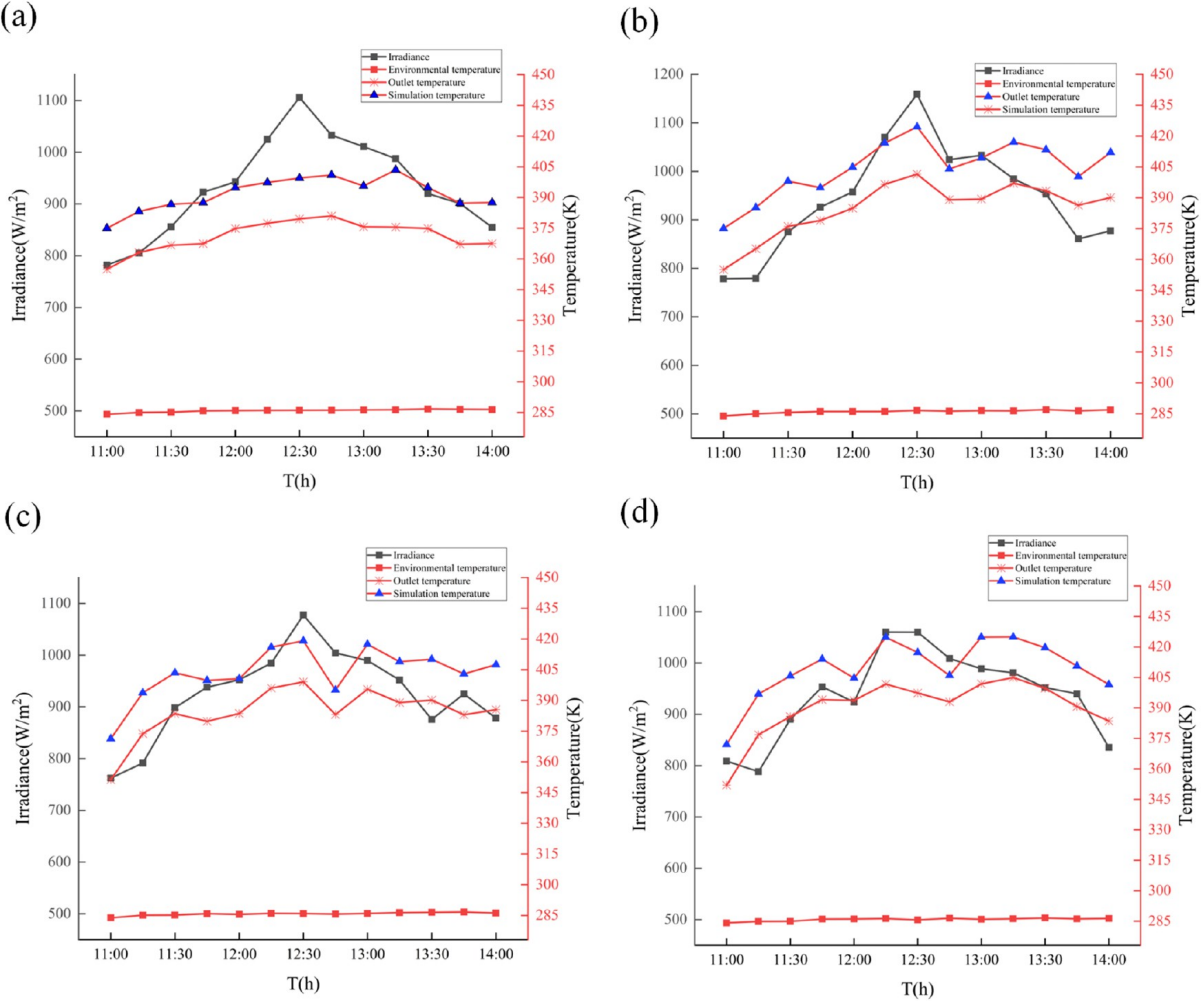
Comparison of exit temperatures for different concentrations of nanofluids: (a) 0 ppm, (b)160 ppm, (c) 280 ppm, and (d) 420 ppm.

As can be seen in Figure 14, the weather was relatively close on all 4 days, with midday ambient temperatures remaining near 10–13 °C. The intensity of solar irradiance also maintained a relatively stable trend with small variations. The measured nanofluid temperature is affected by the solar irradiance variation in the general trend, but the nanofluid temperature profile in the real environment is relatively flat compared to the simulation data in the ideal case, which is strictly affected by the irradiance variation. In addition to this, the nanofluid temperatures under the experiments are all about 20–30 K lower than the simulated cases, which may be affected by other factors in the environment. From the point of view of nanofluids with different concentrations, the change in concentration does affect the amount of heat absorbed by the nanofluid, which increases as the concentration increases for similar solar irradiance and ambient temperature. This trend is also consistent with the simulation results of this study.

**Figure 15:**
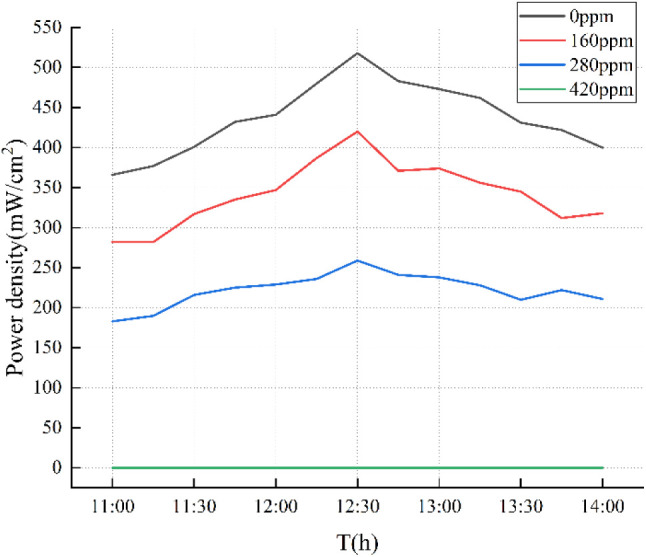
Experimental effects of different concentrations of nanofluids on photovoltaic cells.

The concentration of the nanofluid exerts a substantial effect on the performance of the photovoltaic (PV) cell, with its impact surpassing that of temperature variations. As shown in Figure 15, the experiments were carried out over several consecutive days with similar weather conditions, the results show that changes in solar irradiance directly affect the power density at a specific nanofluid concentration, but the power density of the PV cell is also greatly affected by the nanofluid concentration.

Specifically, for the four nanofluid concentrations tested (0, 160, 280, and 420 ppm), the difference in measured output power density ranged from 121 to 163 mW/cm^2^. These results indicate that as the nanofluid concentration increases, the output power density decreases. This observed trend is consistent with the numerical simulation results, further confirming the validity and applicability of the simulation model in predicting the effect of nanofluid concentration on PV cell performance.

## 4 Conclusion

In the context of efficiently and flexibly utilizing the full solar spectrum, this paper proposes a multifunctional CPV/T system that integrates concentration, frequency splitting, heat transfer, and power generation. Building on this foundation, the optical characteristics and frequency-splitting structure of the combined system were investigated, and the system’s thermal and electrical characteristics were analyzed in relation to the light absorption properties of the frequency-splitting medium. A CPV/T hybrid system that allows for flexible adjustment of the heat-to-electricity ratio by varying the concentration of the frequency-splitting medium is proposed. The key conclusions are as follows:

1 Using the ray tracing validation model, the cylindrical conduit with a flat bottom and concave top achieves optimal spot uniformity at a curvature of 1.19, resulting in a 14% improvement compared to a system without the frequency-splitting component.
2 As the concentration of nanofluid increases from 0 to 80 ppm, the attenuation of light intensity on the cell surface occurs rapidly across various irradiance levels, with the effect becoming more pronounced at higher irradiance. Beyond this point, the light intensity decreases linearly with increasing concentration, approaching zero at a concentration of 420 ppm.
3 As the nanofluid concentration increases from 0 to 420 ppm under different irradiance conditions, the temperature rises by approximately 20 to 25 K. Simultaneously, the cell surface temperature decreases rapidly, particularly under higher irradiance levels. At 1000 W/m^2^ and 0 ppm concentration, the cell temperature reaches a maximum of 364 K, a reduction of 372.61% relative to the cell surface temperature before frequency-splitting. The remaining visible light and a minor portion of infrared light are absorbed during the frequency-splitting process as concentration increases.
4 At the same irradiance, for every 40 ppm increase in nanofluid concentration, the thermal efficiency increases by 3% to 4%. However, the rate of increase diminishes after reaching 300 ppm, with a more modest rise of only 5% as concentration increases from 300 to 420 ppm. The cooling conduit behind the photovoltaic cell absorbs residual heat, enhancing the thermal efficiency by 1.05% compared to a system without heat absorption.
5 In terms of photovoltaic generation efficiency, for every 100 W/m^2^ increase in irradiance, the efficiency improves by 0.12% to 0.40%. When the nanofluid concentration reaches approximately 280 ppm, the overall efficiency peaks. Beyond this concentration, further increases lead to a significant decline in photovoltaic cell efficiency and a rapid drop in overall system efficiency.
6 Experimental measurements of nanofluid temperature and photovoltaic (PV) system power output showed that nanofluid concentration has a significant effect on PV cell performance. The results of the study are consistent with simulations, including the fact that the higher the concentration, the lower the PV power output, thus confirming the potential of regulating the nanofluid concentration to optimize the system output.

## Supporting Information Available

Figure S1: specific shape of the cross-section; Figure S2: ray tracing simulation based on TracPro software; Figure S3: ZnO nanofluid preparation process; Figure S4: absorbance of nanofluids at different concentrations; Figure S5: optimal flow rate under different concentrations and irradiance levels; Figure S6: comprehensive system efficiency under different specular spot ratios; Figure S7: thermal conductivity of nanofluids at different concentrations; Figure S8: heat capacity of nanofluids at different concentrations; Table S9: the equipment used in the experimental setup (PDF)

## Nomenclature

*q*_1_: The heat flux vector
*A*_1_: The mirror’s normal projection area (m)
*T*_0_: The ambient temperature [K]
*A*_1.glass_: The exposed surface area of the duct [m]
*T*_h_: The blackbody temperature [K]
*C*: The speed of light [s/m]
*T*_1.grass_: The temperature of the duct surface [K]
*Cp*: Specific heat of nanofluid [J/kg·k]
*T*_1_: The mean temperature of the mixed fluid [K]
*D*_1_: Nanoparticle diameter [m]
*V*_*OC*_: Open-circuit voltage
*E*_*PV*_: Energy reaching the photovoltaic cell
Δ*E*: Spot uniformity [%]
*E*_PV–opt.loss_: Optical loss in photovoltaic cells
Δ*E*_max_: The maximum irradiance intensity [%]
*E*_el_: Electricity from photovoltaic cells
Δ*E*_ave_: The average irradiance intensity [%]
*FF*: Fill factor of the photovoltaic cell^19^
*α*_PV_: The absorption factor of the photovoltaic cell
*h*: Planck’s constant
ε_PV_: The emissivity of the photovoltaic cell
*h*_PV_: The convective heat transfer coefficient
*ε*_1.glass_: The emissivity of the duct surface
*h*_1_: The convective heat transfer coefficient of the nanofluid
γ_PV_: The intercept factor of the photovoltaic cell
HER: The value of thermal energy relative to electrical energy
η_cref_: The total spectrum reflectance of the mirror
*i*: Light Intensity [W/m^2^]
ηpv: The electric efficiency
*J*_sc_: The short-circuit current of the photovoltaic cell
η_th_: The system’s thermal efficiency
*k*_B_: Boltzmann’s constant
λ: Wavelength (nm)
*k*_P_: Thermal conductivity of the nanofluid
ρ_1_: The density of nanofluid
*K*: The conversion fraction
τ_1_: Nanofluid spectral transmittance
*L*_m_: Thickness of Nanofluid (m)
τ_glass_: The transmittance of the glass surface of the photovoltaic module
*Nb*: Concentration (ppm)
PV: Photovoltaic
*ni*.*zf*: Extinction coefficient of nanoparticles
PT: Photothermal
*ni*_total_: Extinction coefficient of nanofluids
CPVT: Concentrated Photovoltaic-Thermal
*ni*: Extinction coefficients of nanofluids of specific thickness
PVT: Photovoltaic-Thermal
*Q*_*α*_: Absorption efficiency factor^16^
CPC: Compound Parabolic Concentrator
*Q*_f_: The solar energy received in the concentrated area
CPV: Concentrated Photovoltaic
*Q*_g.loss_: the optical losses of the reflector
PCS: Concentrated Spectrum-Splitting
Q*_f_*_1_: Energy reaching the pipeline
ZnO: Zinc Oxide
Q_r.loss_: Duct heat loss
Al_2_O_3_: Aluminum Oxide
*Q*_r.loss-rad_: Radiation loss from nanofluids
AM1.5: Air Mass 1.5 (a standard solar spectrum)
*Q*_r.loss-conv_: Convective losses in nanofluids
GaAs: Gallium Arsenide
*Q*_PV-heat.loss_: Heat loss from photovoltaic cells
TracePro: Optical software used for ray-tracing simulations
*Q*_PV-fluid_: Utilizable waste heat from photovoltaic cells
COMSOL: COMSOL Multiphysics Simulation Software
*Q*_*r*_: Heat gained by piping
UV-1601: Beifen Ruili UV-1601 Spectrophotometer
*Q*_*r*1_: Thermal energy used to generate electricity
