# Supplementary material for: Considering the Performance Study of ZnO Nanofluid at Different Concentrations for the Full-Spectrum Utilization System
Source: ACS Omega. 2025 Apr 16;10(16):16355–69. doi: 10.1021/acsomega.4c10744 (PMC12044469; doi:10.1021/acsomega.4c10744)
Supplement: Supplementary file 1 — ao4c10744_si_001.pdf [file ao4c10744_si_001.pdf]

Considering the performance study of ZnO nanofluid at different concentrations for full spectrum utilization system

Yangjie Zhuang[a], Yizhi Tian[a,*], Min Li[b]

[a] College of Electrical Engineering, Xinjiang University, Urumqi 830017, P.R. China

[b] Key Laboratory of Oasis Ecology of Education Ministry, College of Ecology and Environment, Xinjiang University, Urumqi 830017, P.R. China

*Corresponding author:

Email address: torsionscale@163.com (Yizhi Tian)

Supplementary Note 1: Simulation software introduction

(1) COMSOL Multiphysics is a versatile multi-physics simulation software developed by COMSOL, which is widely used in engineering and scientific research fields. A parametric diagram of the cross-section of the pipe is shown in **Figure S1**, with the inner cavity filled with nanofluid. (2) TracePro is a light simulation software widely used in lighting systems, optical analysis, radiosity analysis and photometric analysis. Developed by Lambda Research Corporation and based on the ACIS solid modeling kernel, it combines real solid models, powerful optical analysis capabilities, powerful data conversion capabilities, and a user-friendly interface, The light simulation of the concentrator device in the software is shown in **Figure S2**.

Supplementary Note 2: Nanofluid Preparation Process

The preparation work for the transmission measurement of the nanofluids shown in **Figure S3** included equipping the nano-fluids with different concentrations of glycerol based zinc oxide, centrifugal agitation, and ultrasonic oscillation.

Figures for Supporting Information

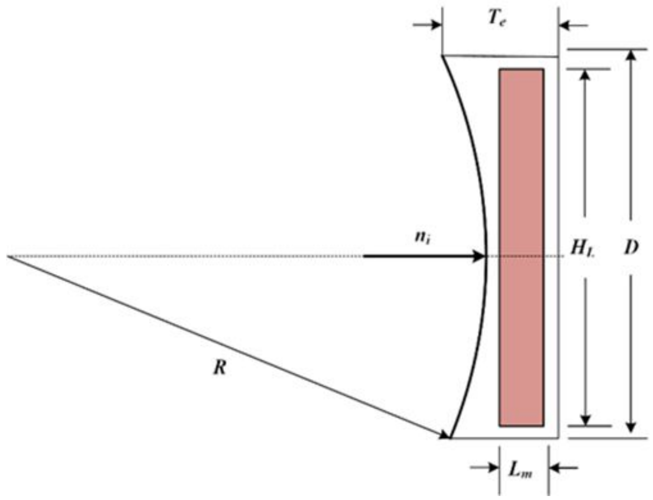

Figure S1. Cross-Section of the Hollow Flat-Bottom, Concave-Cylinder Pipeline.

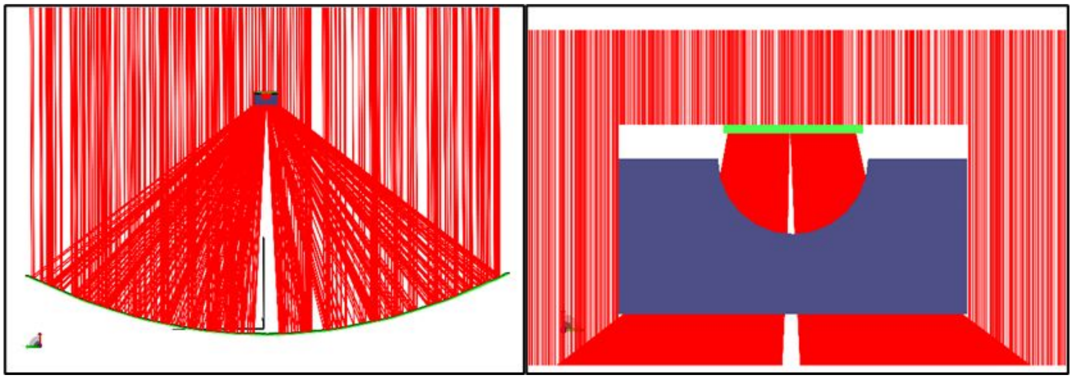

Figure S2. Ray-Tracing Diagram of the Spectrum-Splitting Pipeline.

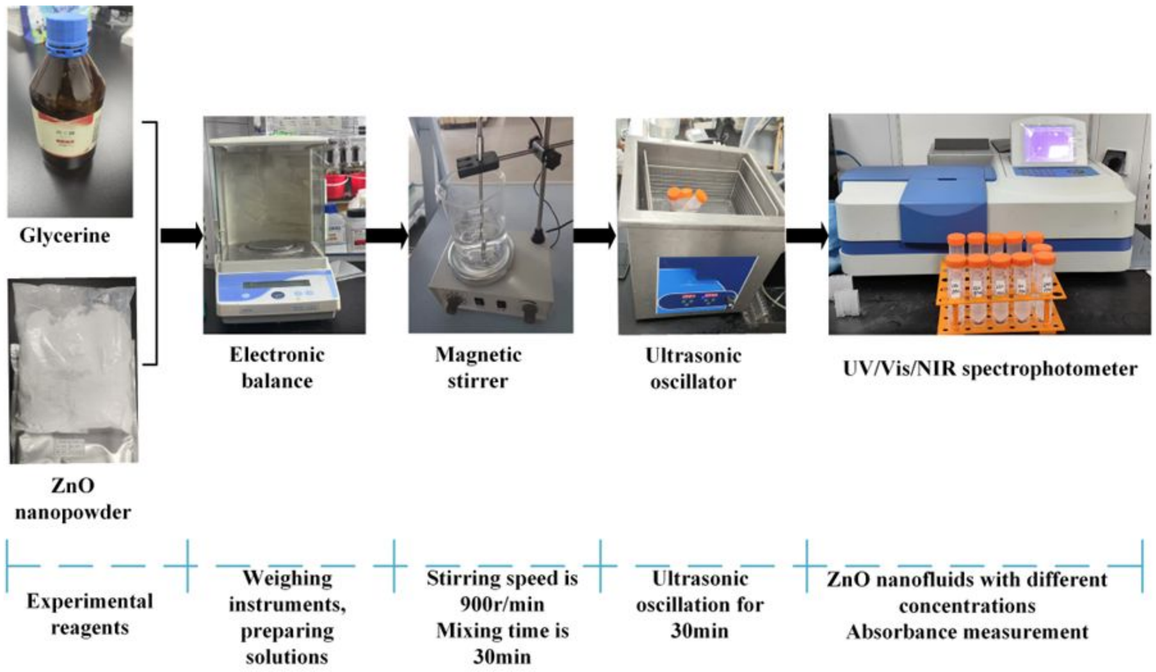

Figure S3. ZnO nanofluid preparation process.

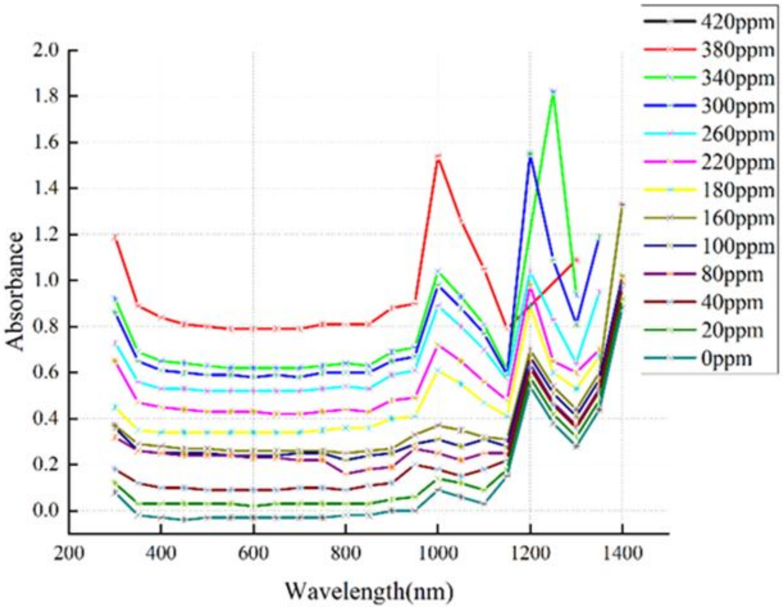

Figure S4. Absorbance of nanofluids at different concentrations.

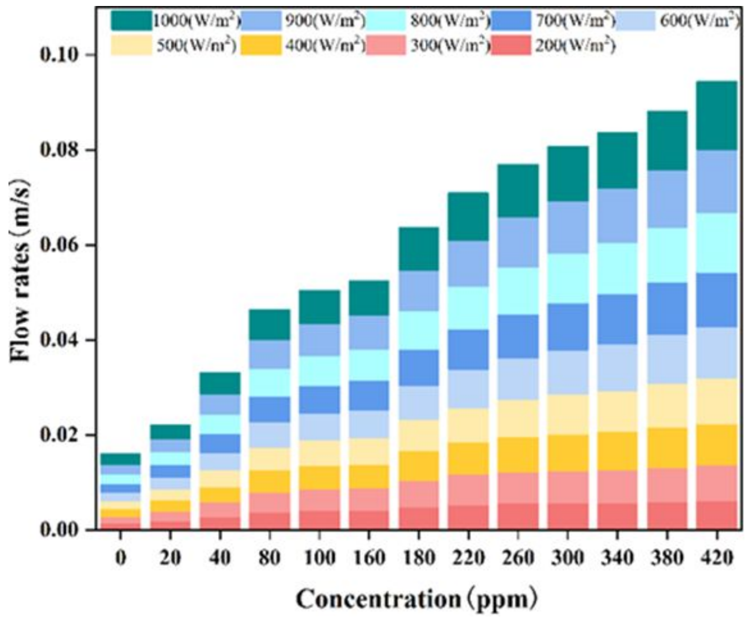

Figure S5. Optimal Flow Rate under Different Concentrations and Irradiance Levels.

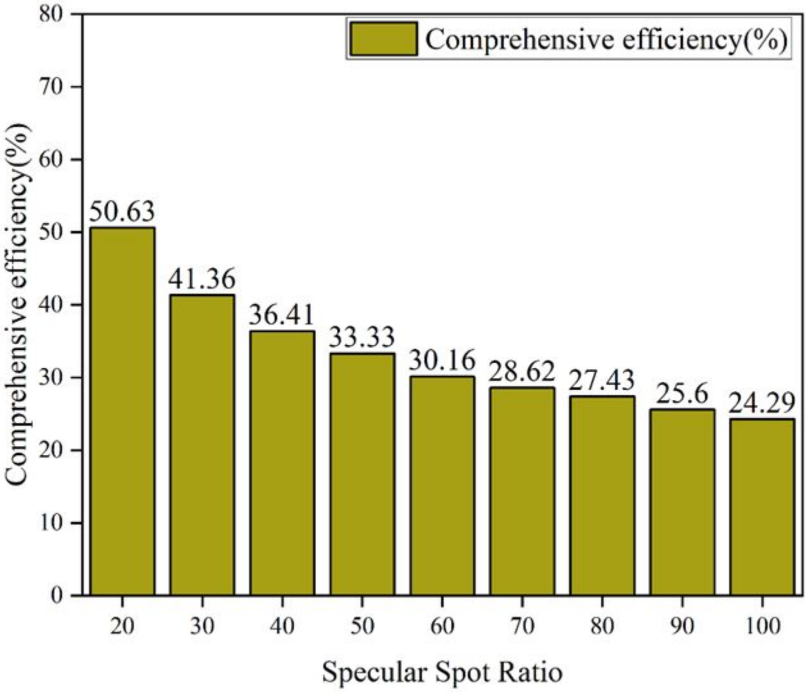

Figure S6. Comprehensive System Efficiency under Different Specular Spot Ratios.

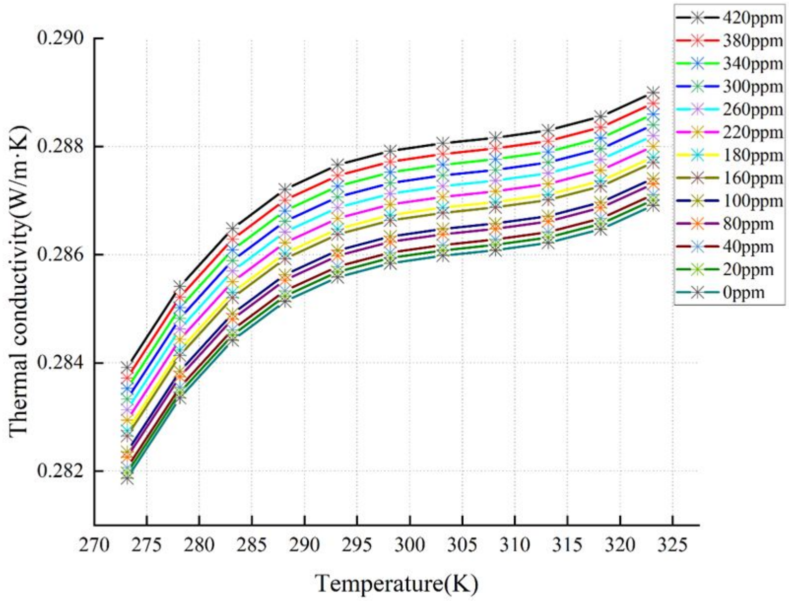

Figure S7. Thermal conductivity of nanofluids at different concentrations.

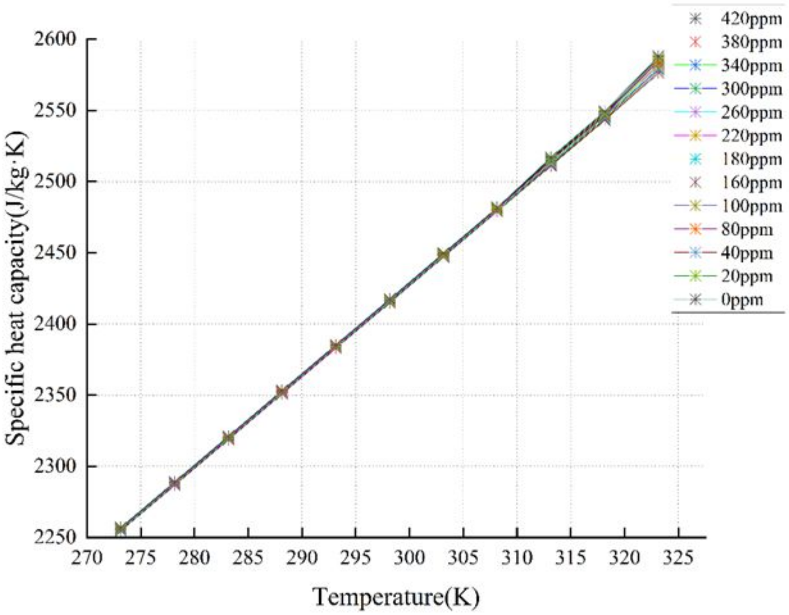

Figure S8. Heat capacity of nanofluids at different concentrations.

Table for Supporting Information

Table S9. The equipment used in the experimental setup.

Parabolic reflector	ALANOD 5011AG
Receiver tube	Quartz glass
Circulating Pump	Suction pump.<br>Product flow: 3-18 L/MIN, Positive pressure: 0.095mPA, Suction: 0.065 mPA
PV cell	4 cells, 10x10mm PV size
Voltage and Current Sensor	PVT801.<br>DC Volts ranges: 200 mV, 2 V, 20 V, 200 V, 1000 V ±(1.0 % rdg +1 dgt) DC ranges: 200 μA, 2 mA, 20 mA, 200 mA ±(1.5 % rdg +1 dgt)
Pyranometer	SM206-SOLAR.<br>Sensitivity Approx.: 0.1 W/m2, Wavelength range: 340-1100 nm, Zero offset-thermal radiation (200 W/m^2): ±1.5W/m^2,<br>Temperature change : less than 0.38 W/m^2/℃
Temperature measurement	RC9550.<br>Range: -55 ℃, 550 ℃ Sensor sensitivity
Multimeter	VC830L.<br>DC Volts ranges: 200 mV, 2 V, 20 V, 200 V, 600 V ±(1.0 % rdg +5 dgt) DC ranges:20μA, 200 μA, 2 mA, 20 mA, 200 mA,10A ±(2.0 % rdg +5 dgt)
